# Patient initiated clinics for patients with chronic or recurrent conditions managed in secondary care: a systematic review of patient reported outcomes and patient and clinician satisfaction

**DOI:** 10.1186/1472-6963-13-501

**Published:** 2013-12-01

**Authors:** Rebecca Whear, Abdul-Kareem Abdul-Rahman, Jo Thompson-Coon, Kate Boddy, Mark G Perry, Ken Stein

**Affiliations:** 1 Peninsula Collaboration for Leadership in Applied Health Research and Care (PenCLAHRC), University of Exeter, Veysey Building, Salmon Pool Road, Exeter, Devon EX2 4SG, England; 2 Peninsula Collaboration for Leadership in Applied Health Research and Care (PenCLAHRC), Peninsula College of Medicine and Dentistry, Veysey Building, Salmon Pool Road, Exeter, Devon EX2 4SG, England; 3 Rheumatology Plymouth Healthcare NHS Trust, Veysey Building, Salmon Pool Road, Exeter, Devon EX2 4SG, England; 4 Public Health, University of Exeter, Veysey Building, Salmon Pool Road, Exeter, Devon EX2 4SG, England

## Abstract

**Background:**

The cost to the NHS of missed or inappropriate hospital appointments is considerable. Alternative methods of appointment scheduling might be more flexible to patients’ needs without jeopardising health and service quality. The objective was to systematically review evidence of patient initiated clinics in secondary care on patient reported outcomes among patients with chronic/recurrent conditions.

**Methods:**

Seven databases were searched from inception to June 2013. Hand searching of included studies references was also conducted. Studies comparing the effects of patient initiated clinics with traditional consultant led clinics in secondary care for patients with long term chronic or recurrent diseases on health related quality of life and/or patient satisfaction were included. Data was extracted by one reviewer and checked by a second. Results were synthesised narratively.

**Results:**

Seven studies were included in the review, these covered a total of 1,655 participants across three conditions: breast cancer, inflammatory bowel disease and rheumatoid arthritis. Quality of reporting was variable. Results showed no significant differences between the intervention and control groups for psychological and health related quality of life outcomes indicating no evidence of harm. Some patients reported significantly more satisfaction using patient-initiated clinics than usual care (p < 0.001).

**Conclusions:**

The results show potential for patient initiated clinics to result in greater patient and clinician satisfaction. The patient-consultant relationship appeared to play an important part in patient satisfaction and should be considered an important area of future research as should the presence or absence of a guidebook to aid self-management. Patient initiated clinics fit the models of care suggested by policy makers and so further research into long term outcomes for patients and service use in this area of practice is both relevant and timely.

## Background

Around 17.5 million people in Great Britain have a chronic condition [1]. Traditionally, those who are managed in the secondary care setting (some conditions are managed in primary care) attend hospital appointments initiated by a physician at regular intervals e.g. every six, nine or 12 months. These appointments commonly occur at a time when a person is feeling well and may result in little change in management of the condition [2]. However, should symptoms recur or worsen it may be difficult to obtain urgent appointments as needed by the patient, not least because the capacity in outpatient departments is devoted to routine follow up [2].

More recently, there has been an emphasis on encouraging people to manage their own care where possible [3]. With no clear pathway defined to achieve this, health services have tried to implement systems to support patients in managing their health condition in a number of ways. UK examples include, the Choose and Book service implemented in 2004 [4] which aimed to provide choice in the timing and location of outpatient appointments, and the Expert Patient Programme established in 2006 [1], which aimed to provide people with long term conditions the skills and support to be able to manage their own condition and make more effective use of healthcare services. In addition, various initiatives have been introduced to promote appointment attendance or avoid unfilled appointments, for example, overbooking appointments, introducing fines for missed appointments and using alerts and reminders. Whilst these systems may help to reduce the number of missed appointments (there were 6.7million missed appointments in 2009/10 reported to cost the National Health Service (NHS) millions of pounds every year) [5] they do not help to make appointment scheduling more responsive to the needs of the patient.

Patient-initiated clinics (PIC) aim to be responsive to patients needs during the fluctuation of their condition and to minimise the likelihood of missed appointments (and hence wasted resources). In PICs, appointments are scheduled by the patient according to their needs unlike traditional clinician led appointments. A patient will phone an advice line where a nurse specialist can provide guidance over the telephone, or if necessary arrange a consultant appointment as soon as possible.

Patient initiated appointment scheduling has been studied in primary care [6-8]. The results of a review in primary care [6] suggested small improvements in waiting times and the rate of missed appointments, but show mixed findings for patient satisfaction. Two other primary care studies suggested there could be substantial improvements in the amount of wasted patient and clinician time with the latter resulting in a reduced cost for care delivery [7,8]. With the increasing focus on health service efficiencies highlighted by the UK Government’s Health and Social Care Bill [9], determining the potential benefits and harms of patient initiated appointments in a broader healthcare setting such as secondary care is timely.

The objective of this study was to systematically review the effects of patient initiated clinics in secondary care for people with chronic or recurrent conditions on patient reported health related quality of life (HRQoL) and psychological outcomes in comparison to care using traditional clinician determined appointment intervals. We were also interested in outcomes that described the acceptability of the PIC service for both patients and clinicians.

## Methods

We conducted the systematic review following the principles published by the NHS Centre for Reviews and Dissemination (CRD) [10]. A protocol for this review was developed in consultation with experts and is available on the PenCLAHRC website (http://clahrc-peninsula.nihr.ac.uk/systematic-review-of-patient-initiated-clinics.php).

### Literature search and eligibility criteria

An information specialist (KB) developed the search strategy in collaboration with a physician and other IS experts to ensure all the appropriate terms were captured. No methods filter was applied to the search strategy in order to capture all available comparative literature. The search was conducted in the following databases: Medline, Embase and Psycinfo (using the OVID interface), the Cochrane Library of Systematic Reviews and CENTRAL, Science Citation Index Expanded, Social Sciences Citation Index, Conference Proceedings Citation Index (via the Web of Science interface) from inception to December 2010 (see Additional file 1: Appendix 1 – online for the full strategy used for Medline). Update searches were conducted from December 2010 to June 2013. We also checked the references of included studies, searched for ongoing research studies and contacted authors and other experts in chronic diseases to identify any further relevant literature.

Studies were included if they reported a comparison (including randomised controlled trials, controlled trials, pre-post and cross-sectional studies) of the effectiveness of patient initiated clinics (the intervention) against routine, clinician-led, follow-up (the control) systems in secondary care for people with chronic or recurrent conditions. To be included studies had to report on at least one relevant patient reported outcome such as psychological outcomes (e.g. anxiety, depression), generic and condition specific HRQoL (e.g. SF36, EQ-5D, IBDQ) or patient or clinician satisfaction. Studies were excluded if there was insufficient information to allow appraisal of study quality, they were set in primary care, they dealt with making a diagnosis (rather than disease management), or included short term acute conditions. No date or language restrictions were applied.

Titles and abstracts were independently screened by two reviewers (JTC, AA or RW) who applied the inclusion and exclusion criteria. Full texts were retrieved for articles that required more in depth application of the inclusion and exclusion criteria. All full texts were independently reviewed by two reviewers (RW and AA) and discrepancies were resolved by a third reviewer (JTC or KS) where necessary.

### Data collection

Data extraction was conducted by two reviewers (RW and AA) and checked by a third reviewer (JTC) using a standardised data extraction form (see Additional file 1: Appendix 2). The data extracted included information on the quality of the study (based on the guidelines from the Centre for Reviews and Dissemination [10]) and information on the participants, intervention and control descriptions, outcomes and outcome measures as well as the results (Additional file 1: Appendix 2).

### Data synthesis

We used narrative synthesis to summarise and discuss the quantitative results of the included studies following the principles described in the Economic and Social Research Council [11] and the Centre for Reviews and Dissemination [10] guidelines. Any qualitative data that were reported by the included studies were reported separately. Meta-analysis was inappropriate due to the small number of studies and participants, and the heterogeneity in participants, their diagnoses and the outcomes measured between the studies.

## Results

**Figure 1:**
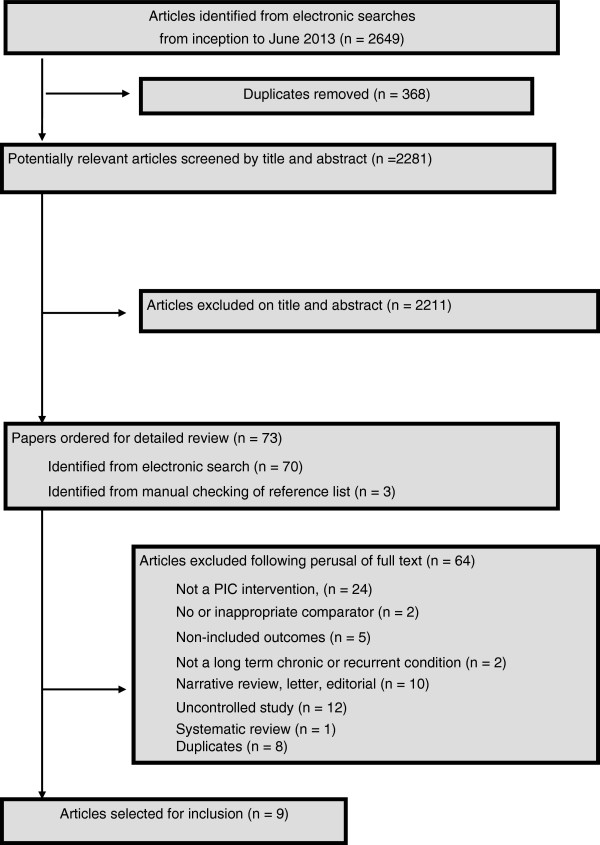
Identification and selection of studies for the review.

Figure 1 summarises the identification and selection of studies. Seven studies (nine articles) (n = 1,655 people; 41% male) met the inclusion criteria [12-20], six were identified by electronic searches and one through contact with experts [13]. Six included studies were randomised controlled trials and one was of a cross-sectional design [20]. One study was reported in three articles at different stages of follow-up [17-19]. In total 2,550 (including 368 duplicate) studies were excluded. Reasons for exclusion at the full text stage are shown in Figure 1.

### Study characteristics

The included studies measured patient reported HRQoL, satisfaction and psychological outcomes, using a range of tools, across three disease areas – breast cancer [12,13] (BC), inflammatory bowel disease [14-16] (IBD) and rheumatoid arthritis [17-20] (RA). All studies were conducted in the UK. A total of 1,655 people over the age of 16 years were studied (275 with breast cancer, 1,083 with inflammatory bowel disease, and 297 with rheumatoid arthritis). Participants were followed up for between 12 and 72 months.

**Figure 2:**
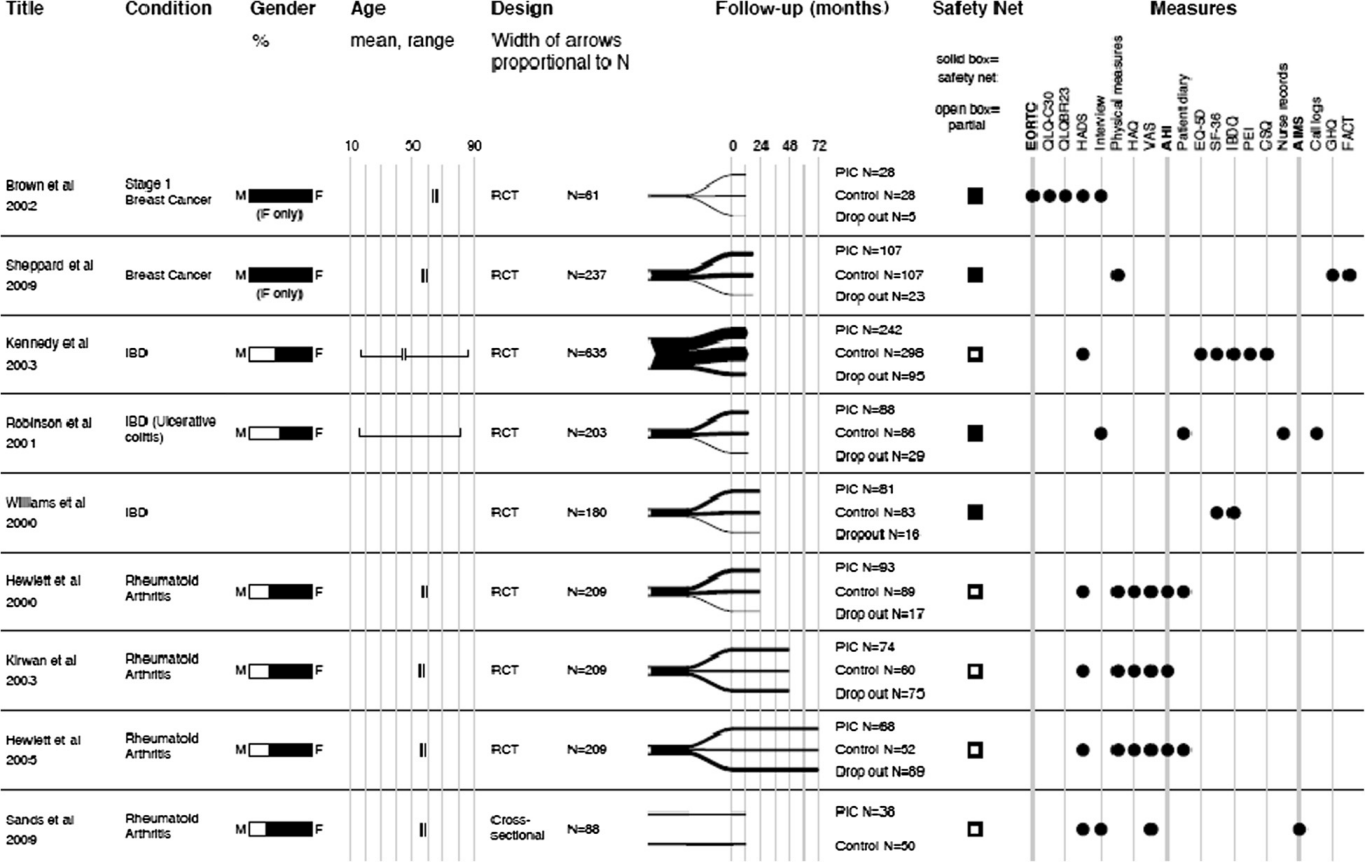
Characteristics of included studies.

The approaches used in the patient initiated clinics were broadly similar across the studies with the main access point being a telephone number through which the patient could request clinical advice and, if required, arrange an appointment to see a clinician. In three studies [14,15,20] people in the intervention group had an initial appointment with a consultant during which a ‘guidebook’ was also used and given to the patient. The ‘guidebook’ contained information to enable the patient to self-manage their condition and a care management plan (Additional file 1: Table S1 online). Eligibility for inclusion in the studies was carefully described in all the papers. In the BC studies, only people with early stage (I or II) disease with no clinical signs of recurrence were included. Inclusion criteria for the IBD studies included having stable, mildly active, established (not newly diagnosed) disease and no other conditions requiring follow-up. Any patient with RA was eligible for inclusion in the RA studies. Four articles collected a mixture of qualitative and quantitative data [12,14,15,20] and five collected only quantitative data [13,16-19]. Figure 2 details the characteristics of the included studies.

### Study quality

Additional file 1: Table S2 (online) summarises the quality of the included studies. Although, most of the studies were randomised controlled trials the quality of reporting was poor and inconsistent. For example, very few of the studies reported the method of randomisation or whether the outcome measures used had established reliability and validity. Very few reported intention-to-treat (ITT) analyses and just over half reported having more than 80% of the participants remaining at follow-up. Furthermore, there was no reporting of the fidelity with which the intervention had been implemented. Most studies reported that the rate of attrition was not significantly different between the intervention and control groups. However, the influence of participant attrition was not adequately recorded or described. Some aspects of the studies were reported well including: details of sample size calculations and baseline participant characteristics. The methods used and reported by Robinson and colleagues [15], Kennedy and colleagues [14], Sheppard and colleagues [13] and Hewlett and colleagues [18,19] suggest lower potential for bias in their results (see Additional file 1: Table S2 - online).

Qualitative data were reported in some studies [12,14,15,20]. These data were retrieved from interviews and patient diaries. Some of the qualitative research was conducted well with clear objectives and detailed analyses [14,20]. However, in others [12,15], there was sparse interpretation of data and little consideration of the limitations associated with the method of data collection.

Following the general framework for narrative synthesis [11], an outline logic model for the theory behind both the traditional and PIC appointment systems is illustrated in Additional file 1: Figure S1 (online). Following this the findings on patient reported HRQoL, psychological and patient and clinician satisfaction outcomes are reported by disease area. Subsequent sections of the results report brief summaries of qualitative findings and use them to explore relationships between characteristics of the intervention and the results.

### Patient quality of life and psychological outcomes

#### Breast cancer

**Figure 3:**
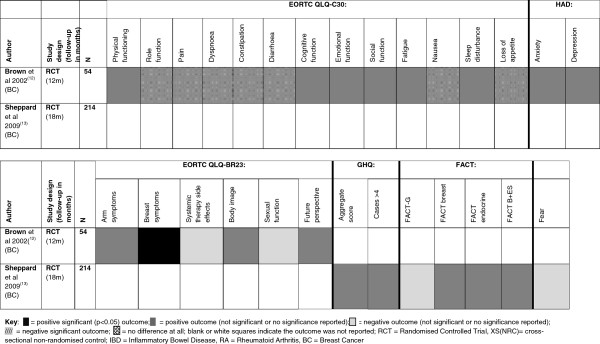
**Patient reported outcomes chart (breast cancer) **[12,13]**.**

Brown and colleagues [12] reported 21 patient reported psychological and HRQoL outcomes (EORTC QLQ-C30 – European Organisation for Research and Treatment of Cancer Quality of Life Questionnaire, EORTC QLQ-BR23, HAD); a further seven outcome measures were reported in the study by Sheppard and colleagues [13]. The results from Brown and colleagues [12] suggested that people in the intervention group reported significantly reduced breast symptoms at 12 months in comparison to the control group (p = 0.024). They also reported that the intervention group tended to have better cognitive function at 12 months, improved sleep patterns, reduced arm symptoms, body image and an improved future perspective than those in the control group but the differences were not significant. Sheppard and colleagues [13] also reported reduced breast symptoms at 18 months (on the Functional Assessment of Cancer Therapy – FACT HRQoL measure) in the intervention group as compared to the control group, although the difference between the two groups was not significant. Systemic therapy side effects and sexual function at 12 months (as reported on the breast cancer specific HRQoL measures the EORTC QLQ BR23) [12]; and total scores for fear at 18 months [13] were worse for the intervention group compared with the control group although the differences were not statistically significant. See Figure 3 and Additional file 1: Appendix 3 for details.

#### Inflammatory bowel disease (IBD)

**Figure 4:**
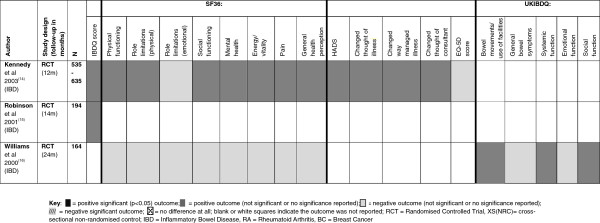
**Patient reported outcomes chart (IBD) **[14-16]**.**

In the three studies of people with IBD, 19 patient reported psychological and HRQoL outcomes (SF36, EQ-5D, HADS, IBDQ) were reported, several of these were shared across the three IBD studies but in some cases findings were contradictory. Kennedy and colleagues [14] reported that people in the intervention group had better mental health scores at 12 months than the control group, although Williams and colleagues [16] reported the reverse at 24 months. Kennedy and colleagues [14] also reported more positive scores in the general health perception of people in the intervention group for which again Williams and colleagues [16] find opposing results at 24 months. Furthermore, Kennedy and colleagues [14] reported that the intervention group were more likely to have changed the way they think about and manage their illness, and to change the way they think about their consultant (see Figure 4 and Additional file 1: Appendix 3 for details).

#### Rheumatoid Arthritis (RA)

**Figure 5:**
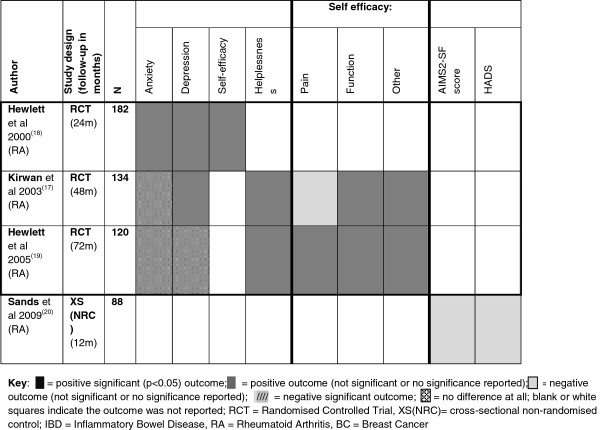
**Patient reported outcomes chart (RA) **[17-20]**.**

In the studies on RA nine patient reported psychological and HRQoL outcomes (HADS, AIMS2-SF) were reported, several of which are shared across the included studies. Self-efficacy and helplessness are two areas only reported in the RA studies. Kirwan and colleagues [17] suggest that people in the intervention group reported feeling less helpless than those in the control group at 48 months (change in scores -0.2 vs. 1.0). At 72 months the intervention group still fares better but both groups have started to feel more helpless than at baseline (change in scores 0.5 vs. 1.0) [19]. At both 48 and 72 months the change in helplessness scores for the control group was *clinically* (but not statistically) significant from baseline. This was not the case for the PIC group although the range of score differences for this group did include a *clinically* (but not statistically) significant level of change (defined by the Arthritis Helplessness Index –AHI as a 1 point change in score). For measures of self-efficacy, the people in the intervention group reported greater levels of self-efficacy than the control group at all stages of follow-up [17] (see Figure 5 and Additional file 1: Appendix 3 for details).

### Outcomes shared across disease areas

Eleven types of patient reported outcomes were shared across more than one disease area: physical function, role function, pain, emotional function, social function, fatigue, anxiety, depression, patient satisfaction and confidence, GP (general practitioner) satisfaction and confidence and patient HRQoL. In most cases the studies have used disease specific instruments to measure these constructs, but it is possible to make some comparisons regarding the general influence of the intervention on patients’ well-being.

The impact of the intervention (PIC) on perceived physical functioning is reported to be positive at 12 months in those with breast cancer or IBD and at 48 and 72 months in those with RA (as measured by the SF36 and EORTC QLQC-30) [12,14,19]. However, Williams and colleagues [16] suggested that this does not continue at 24 months for people with IBD. The results for role function (a subscale relating to the patient’s ability to complete work or household jobs) were rather more negative, with most of the outcomes across the same three studies reporting worse levels of role functioning in the intervention group than the control group at all stages of follow-up.

Patient reported pain levels across disease area varied greatly. There were no reported differences between the control and intervention groups in one breast cancer study [12]. Kennedy and colleagues [14] reported improved levels of pain in the IBD intervention group at 12 months (m = 69.5 SD27.6 vs. M = 67.1 SD23.6, p = 0.22). However, by 24 months this relationship had reversed (mean difference -2.5, 95% CI 5.0,-10.5) [16]. This is also reported in the RA studies until 48 months but at 72 months Hewlett and colleagues [19] reported that pain levels have again improved in the intervention group more than in the control group, although these differences were not significant.

Emotional and social function outcomes also show mixed results across disease areas. Brown and colleagues [12] reported that both social and emotional functioning was better in the intervention group with breast cancer than the control group at 12 months. However, Williams and colleagues [16] reported the reverse to be true at 24 months follow-up. Fatigue, energy and vitality offered similar results across the breast cancer and IBD studies [12,14,16]. Self-efficacy of function and pain was measured only in the RA studies by the Arthritis Self-Efficacy Scales (ASES) [17,19]. The impact of the intervention (PIC) in comparison to the control group on perceived self-efficacy of functioning is reported to be positive at 48 and 72 months in those with RA.

More consistent results can be seen in anxiety and depression scores from people with breast cancer or rheumatoid arthritis. Brown and colleagues [12] reported that the people with breast cancer in the intervention group had better anxiety and depression scores than the control group at 12 months (P = 0.069), although scores for both groups remained largely in the ‘healthy’ range on this measure. This is also shown by Sheppard and colleagues, [13] who reported slightly better scores on psychological morbidity (fear) in people with breast cancer who are in the intervention group (m = 5.6 vs. m = 5.0). In people with RA Hewlett and colleagues [18] reported similarly positive impacts of PIC on anxiety and depression at 24 months, these findings were maintained at 48 months [17]. However, by 72 months the RA intervention group reported similar levels to the control group. There were no *clinically* significant differences between the two groups on these scores at 24 months although the control group had borderline disorder scores for anxiety. By 48 months anxiety for both groups had been pushed into the borderline range but there remained no clinically significant differences between the groups, and this remained the same at 72 months. These data suggest that people’s psychological state is either similar or better with patient initiated care.

Finally, four studies reported an overall HRQoL score for their patients. Robinson and colleagues [15] suggested that people with IBD in the intervention group reported higher levels of disease specific HRQoL than the control group at 14 months (m = 189 vs. m = 183, p = 0.16), as did Kennedy and colleagues [14] (m = 172 vs. m = 168, p = 0.45). However, Sands and colleagues [20] and Sheppard and colleagues [13] suggested the opposite for disease specific HRQoL in people with RA and BC at 12-18 months. Interestingly, Kennedy and colleagues [14] also found that their IBD intervention group were more likely to score *worse* in the more general EQ-5D HRQoL questionnaire than the control group (m = 0.707 vs. m = 0.69). None of these differences were statistically or *clinically* significant.

### Acceptability

#### Patient satisfaction

**Figure 6:**
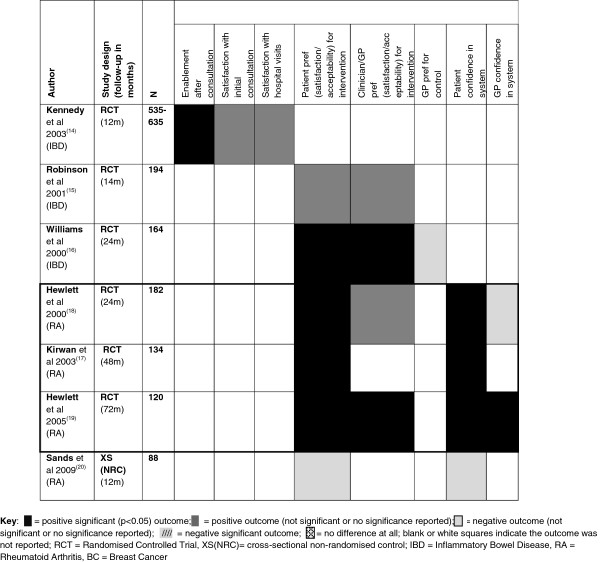
**Patient and clinician satisfaction/acceptability **[14-20]**.**

Acceptability of, satisfaction with, and confidence in the intervention was not recorded in the studies reporting on people with breast cancer. The IBD studies reported that those people in the intervention group were more likely to find their follow-up system acceptable than the control group following traditional appointment systems [15]. For example, patients with IBD in the intervention group were marginally more satisfied with their initial consultation than the control group (m = 65.4 SD12.0 vs. m = 62.1 SD12.3, p = 0.09) [14]. Those people’s preferences for the intervention were also higher than the control group preferences [14,16]. In one study [14], IBD patients in the intervention group were reported to be significantly more confident in themselves than the control group after the initial consultation (m = 4.0 SD3.9 vs. m = 3.0 SD3.9, p = 0.026). Those with RA in the intervention group also generally reported significantly (p < 0.001) greater levels of satisfaction and confidence in the intervention than the control group [17-19]. However, Sands and colleagues [20] reported the reverse to be true although differences were marginal (m = 7.15 SD3.41 vs. m = 7.17 SD3.02, p = 0.990) (Figure 6).

#### Clinician satisfaction

Acceptability to clinicians is clearly important for implementation of any model of health care. Positive results were shown across conditions and were maintained at different stages of follow up (Figure 6). Hospital clinicians and primary care physicians for people with IBD reported the intervention was acceptable (if not preferable) [15,16]. Similarly, hospital and community clinicians in the RA studies reported significantly higher levels of satisfaction (median change 8.4 vs. 7.5, p = 0.005) and confidence (median change 8.4 vs. 8.0, p = 0.04) with the intervention than the control group at 72 months.

### Exploration of relationships

Additional file 1: Figure S1 (online) describes one possible theory about how PICs could lead to a more effective and satisfactory service without harming patients, additional boxes in red are where the studies included in this review may inform the future implementation of PICs. The following section highlights several factors mentioned across the studies and uses the available qualitative evidence (from the included studies [12,14,15,20]) to explore them further.

Although open access appointments were generally reported as a beneficial change to care, there was variability in the way the approach was provided or used. The clinics reported by Kennedy and colleagues [14] retained fixed appointments for those who had unstable IBD disease or who were newly diagnosed and needed the reassurance of regular meetings. This preference for being able to select ‘appropriate’ IBD patients to use the PIC system was also highlighted in the qualitative data collected from clinicians by Robinson and colleagues [15]. Support for this approach could, arguably, be seen across RA and BC with reports of people mis-using the PIC system by making appointments through NHS direct^a^ to access healthcare [14,20] and by a preference to have some degree of scheduled check-up appointments [12] – both of which were influenced by the patients perspective of the role of their consultant.

Further support for the idea of ‘eligibility’ to use the PIC system comes from the characteristics of patients who described themselves as satisfied or dissatisfied with the PIC system. The findings from Kennedy and colleagues [14] suggested that those who were not satisfied with the intervention generally had unstable or chronic IBD symptoms, and had fewer support resources at home. In contrast, those who reported more satisfaction with the intervention tended to be more able to regulate and normalise their illness at work [14]. The results from Kennedy and colleagues study also show more positive influences of the PIC intervention on patient reported HRQoL and psychological outcomes which may have been influenced by the use of the ‘eligibility’ concept, though it is impossible to see the true impact of ‘eligibility’ from these studies alone.

Use of a guidebook or an initial consultation in the PIC intervention might have been expected to produce more significant positive results for the outcomes of interest in this review. Where a guidebook was used it was reported to have increased the knowledge of all patients (IBD) [14]. For most people, the guidebook provided reassurance and help as it enabled sharing of information with family members to inform and raise awareness. However there were no obvious differences in the results across health conditions, which may be a reflection of the reported mixed use of the guidebook in consultations [14]. Some people reported that consultations were rushed and the self-management plan was not recorded which is also reflected in some clinicians concerns that the training (guidebook) did not take account of the time-pressured environment they work in. In these cases, people reported feeling intimidated by the clinician. Clinicians in Kennedy and colleagues [14] study reported a generally positive perspective on the guidebook, that it was particularly helpful in making the consultation more personalised which created a positive environment to encourage self-management. They also acknowledged that being developed in collaboration with patients made the guidebook a more useable tool for conveying information. Despite this the conflict with time may greatly impair the potential influence of this tool. Within the IBD studies Williams and colleagues [16] reported more negative impacts of the PIC intervention on a general measure of HRQoL than Kennedy and colleagues [14]. This could be a reflection of the lack of an initial consultation or any written guidance reported in Williams and colleagues study. However, the real impact of the guidebook and initial consultations on outcomes cannot be confirmed by these studies.

One key aspect which might affect the satisfaction with any appointment based intervention is the relationship between the patient and their consultant/clinician. Those who reported a high regard for their consultant also felt that the PIC option reinforced their confidence in self-managing their condition [12,20]. Whereas those who were dissatisfied included those who felt intimidated or emotionally let down by their consultant when their condition worsened [14]. The importance of this relationship is also highlighted by people with RA in Sands and colleagues [20] study who did not receive a guidebook as part of their intervention but did report being able to get a good level of information from the consultation. Furthermore, an important aspect of the patient-centered consultation for patients was continuity of care. Many were not concerned if they had to see a different doctor as long as they were deemed competent [14,20]. However, there were complaints about missing case-notes and communication difficulties [14], both of which would be particularly important to the smooth running and safety of a PIC.

The development of a good patient-consultant relationship will depend on a number of things, not least the amount of time allowed for the consultation itself and the attitude of the patient and clinician towards each other. Some of the training provided for consultants in Kennedy and colleagues [14] study emphasised spending more time with the patient to allow a discursive and open consultation. Whilst some clinicians found it useful to have somewhere to write down the agreed management plan, others felt that the use of a management plan to form part of a mutual discussion was unrealistic and reported that few (if any) patients had actually attempted to change the management plan with the consultant [14].

## Discussion

We identified seven studies of the impact of patient initiated clinics in secondary care on psychological, HRQoL and satisfaction outcomes in people with breast cancer, IBD and RA. Overall, there were few differences in psychological and HRQoL assessed in those initiating outpatient follow-up compared with standard outpatient care across all three conditions. There were generally positive trends in favour of the PIC system despite the diversity of the conditions. Additionally, patient and clinician satisfaction was often significantly greater with PICs than with regular appointment scheduling for both IBD and RA (these outcomes were not measured in the BC studies). Using the logic model in Additional file 1: Figure S1 online it is possible to see how the PIC system might benefit both patients and the NHS but further research in the efficacy of this approach is needed.

The identified studies imply that intervention fidelity is important as in instances where the intervention was not delivered as intended, or where communication was poor, there was limited facilitation of self-care. The most satisfied individuals were those who had a good relationship with their consultant and who received sympathy and reassurance during their consultations, who felt listened to and given an opportunity to ask questions [14]. Dissatisfaction among patients highlighted challenges for delivering the PIC service, such as rushed appointments, overbooked clinics, and problems arranging appointments around early morning routines [14]. The guidebook may play a role in helping to develop good relations between consultants and patients but further research is needed to explore the reality of using a guidebook in this setting and its implications for the consultant/patient relationship.

Although most of the included studies were randomised trials, the poor reporting of the methods used, the unclear reliability and validity of the some of the tools used for measuring outcomes, and the wide range of follow-up periods, means that there are uncertainties with how the results can be interpreted. The fidelity of implementation of the intervention was not specifically measured in any study. This is a significant limitation of the evidence base as it means there is no knowledge regarding how easy a PIC might be to implement or how effective it might be in particular conditions. A further limitation is that all included studies were conducted in the UK. This may limit the ability to apply our findings to an international healthcare context and may highlight a problem with identifying relevant studies that are not described in a way that could be identified by our search. Taking into account the eligibility of patients in specific conditions to be safely included in a PIC system, it is reassuring that there was little difference in attrition rates between intervention and control groups. It is important to note the potential influence that being in a research study might have had on the effectiveness or acceptability of the PIC. For example, in Hewlett and colleagues studies [17-19] all participants were assessed every three months (for the first 24 months) via a questionnaire and those who failed to complete the questionnaire or who showed evidence of clinical deterioration were followed up by phone. This level of attention would not happen in a real setting. A further limitation of the synthesis is that most of the qualitative evidence stems from one paper [14] which may limit the generalisability of the findings across other health conditions.

This is the first systematic review of PICs in secondary care and benefits from a well designed and extensive search of literature sources. We have been able to compare and contrast the effects of the intervention in a range of chronic conditions, although we were surprised not to find evidence of this type of appointment scheduling for other fluctuating chronic conditions such as asthma, eczema and Parkinson’s disease. Although, we identified, an ongoing trial investigating the use of outpatient on-demand clinics for people with Chronic Obstructive Pulmonary Disease in the Netherlands (http://clinicaltrials.gov/ct2/show/NCT00556816) it is possible that this type of approach to scheduling follow-up takes place in other specialties in a less formal or more localised way, for example as a hospital audit [21].

Recent guidelines for the management of RA and IBD in adults suggest that patients should be able to access a specialist as soon as possible if their condition worsens [22-24]. The guidelines for RA management also highlight the need to support systems that promote self-management of care and treatment [22]. The guidelines state that people with stable RA should be able to obtain appointments at a frequency (and location) suitable to their needs [22]. The guidelines for IBD state that services should enable patients to access specialist care within five days of a relapse and should also have access to a telephone support service [24]. The results of this review suggest that a PIC approach may be an effective and safe way to meet these aims, and raises the question ‘Why have PICs not been implemented more widely across secondary care and other health conditions?’

Lack of implementation may be due to a number of factors. Perhaps the lack of detail about how PICs are implemented (although some exist [25]) and how the context influences the effectiveness and safety of the system has left some apprehension within clinicians as to the applicability of PIC for *their* patients. In the past regular scheduled appointments have helped identify other health conditions within patients and so it may be possible that the alternative scheduling system may lead to under-diagnosis and increased costs in the longer term. This should be monitored in future evaluations of the PIC system. There is also likely to be a need to change administrative support to enable PICs to function efficiently. For example, a patient might decide in conversation with a nurse by telephone that an appointment is required, and currently the nurse specialist may find barriers, such as location of administrators, in booking an appointment. This geographical distance can make for poor team working as people working in the chain may not see the overall benefit of having a responsive system. Additionally, ring fenced appointments need to be kept back for people requiring a PIC. In some institutions the current NHS secondary care appointment system is complex and makes keeping “free” appointments difficult.

Furthermore, resources are required up front to enable patient education to confidently facilitate transfer of care to a PIC. The ‘Payment by Results’ (PbR) system used by the NHS for delivering chronic disease care has the unintended consequence of encouraging clinicians and care providers to see patients more frequently than they may require. An emphasis on patients receiving self-management guidance in the context of a PIC may mean that clinicians see fewer patients per day. However, despite these concerns there is evidence that there can be overall cost savings by using a PIC system [26] although further research is needed.

In addition to organisational barriers to implementation, personal or professional beliefs may mean clinicians are not willing to change their practice. It is important that selection of patients to access care via a PIC system is undertaken carefully. PIC outpatient follow-up is only possible in circumstances where patients or reliable carers can easily identify changes in health status and then successfully contact health services. PICs may not be appropriate for some people with mental health illness or significant social isolation.

Future research to confront and explore these concerns is needed, to enable increasingly safe, effective and efficient implementation of PICs. Mixed methods research exploring patient outcomes across different health conditions, healthcare systems and countries is needed to clearly establish any potential harms of the PIC system, and thus influence its uptake.

## Conclusions

Conservative estimates suggest that outpatient follow-up in some long-term and recurrent conditions may have similar HRQoL and psychological outcomes to traditional clinician-led outpatient follow-up. We found no evidence of harm to patients associated with patient initiated follow-up and patient and clinician satisfaction favours the PIC model. However, PICs are only appropriate in diseases where it is easy for the patient to identify when there is a clinical problem requiring advice, and where they are able to initiate access to medical services. There is also a need to establish the most effective methods of PIC implementation, for example the use of guidebooks may or may not be a key characteristic (something the current research literature cannot resolve). Equally, the importance of the relationship between patient and consultant needs to be explored in relation to the fidelity of implementation and effectiveness of PIC. Reasonable safety nets are also required to ensure safety throughout the care pathway.

## Endnote

^a^NHS Direct is a service where the public can phone the NHS for advice regarding treatment for symptoms or injury. An NHS nurse responds to the call and offers advice.

## Competing interest

Dr Mark G Perry is currently involved with implementing and evaluating a patient initiated clinic (PIC) for people with Rheumatoid Arthritis at Plymouth Healthcare NHS Trust.

## Authors’ contributions

RW was involved in developing the protocol, screening, data extraction and analysis and was involved in drafting the paper. AA was involved in developing the protocol, screening, data extraction and editing the paper. JTC was involved in developing the protocol, screening, data extraction, interpretation of the results and drafting and editing the paper. KB developed and conducted the search strategy, retrieved the texts and contributed to editing the paper. MP was involved in developing the protocol, interpreting the results and in drafting and editing the paper. KS was involved in developing the protocol, interpreting the results and drafting and editing the paper. All authors read and approved the final manuscript.

## Pre-publication history

The pre-publication history for this paper can be accessed here:

http://www.biomedcentral.com/1472-6963/13/501/prepub

## Supplementary Material

Additional file 1 **Appendix 1.** Search strategy. **Table S1**. Characteristics of included studies. **Table S2**. Patient reported outcomes chart (breast cancer). **Table S3**. Patient reported outcomes chart (IBD). **Table S4**. Patient reported outcomes chart (RA). **Table S5**. Patient and clinician satisfaction/acceptability chart. Click here for file
