# Supplementary material for: Patient initiated clinics for patients with chronic or recurrent conditions managed in secondary care: a systematic review of patient reported outcomes and patient and clinician satisfaction
Source: BMC Health Serv Res. 2013 Dec 1;13:501. doi: 10.1186/1472-6963-13-501 (PMC3879028; doi:10.1186/1472-6963-13-501)
Supplement: Additional file 1 — Appendix 1. Search strategy. Table S1. Characteristics of included studies. Table S2. Patient reported outcomes chart (breast cancer). Table S3. Patient reported outcomes chart (IBD). Table S4. Patient reported outcomes chart (RA). Table S5. Patient and clinician satisfaction/acceptability chart. [file 1472-6963-13-501-S1.docx]

Online Documents

**Appendix 1 – Search strategy**

The search strategy prepared for Medline MEDLINE(R) In-Process & Other Non-Indexed Citations and Ovid MEDLINE(R) <1950 to Present> (searched via the OVID SP interface). Strategy adapted for other databases.

Searched: 26-11-10

1. (open access adj5 (follow up* or followup*)).ti,ab. (15)

2. (open access adj5 (check up or checkup or check ups or checkups)).ti,ab. (0)

3. (open access adj5 appointment*).ti,ab. (18)

4. (open access adj5 (clinic or clinics)).ti,ab. (88)

5. (open access adj5 (out patient* or outpatient*)).ti,ab. (19)

6. (Patient* adj5 direct access).ti,ab. (106)

7. (Patient* adj3 initiate* adj5 (follow up* or followup*)).ti,ab. (44)

8. (Patient* adj3 initiate* adj5 (check up or checkup or check ups or checkups)).ti,ab. (0)

9. (Patient* adj3 initiate* adj5 appointment*).ti,ab. (5)

10. (Patient* adj3 initiate* adj5 (clinic or clinics)).ti,ab. (10)

11. (Patient* adj3 initiate* adj5 (out patient* or outpatient*)).ti,ab. (18)

12. (Patient* adj3 led adj5 (follow up* or followup*)).ti,ab. (20)

13. (Patient* adj3 led adj5 (check up or checkup or check ups or checkups)).ti,ab. (0)

14. (Patient* adj3 led adj5 appointment*).ti,ab. (2)

15. (Patient* adj3 led adj5 (clinic or clinics)).ti,ab. (21)

16. (Patient* adj3 led adj5 (out patient* or outpatient*)).ti,ab. (8)

17. (Patient* adj3 request* adj5 (follow up* or followup*)).ti,ab. (58)

18. (Patient* adj3 request* adj5 (check up or checkup or check ups or checkups)).ti,ab. (2)

19. (Patient* adj3 request* adj5 appointment*).ti,ab. (28)

20. (Patient* adj3 request* adj5 (clinic or clinics)).ti,ab. (26)

21. (Patient* adj3 request* adj5 (out patient* or outpatient*)).ti,ab. (11)

22. (self* adj1 referr* adj5 (follow up* or followup*)).ti,ab. (13)

23. (self* adj1 referr* adj5 (check up or checkup or check ups or checkups)).ti,ab. (1)

24. (self* adj1 referr* adj5 appointment*).ti,ab. (1)

25. (self* adj1 referr* adj5 (clinic or clinics)).ti,ab. (47)

26. (self* adj1 referr* adj5 (out patient* or outpatient*)).ti,ab. (15)

27. (patient adj5 (led or request* or initiate*) adj5 review).ti,ab. (25)

28. 1 or 2 or 3 or 4 or 5 or 6 or 7 or 8 or 9 or 10 or 11 or 12 or 13 or 14 or 15 or 16 or 17 or 18 or 19 or 20 or 21 or 22 or 23 or 24 or 25 or 26 or 27 (553)

29. "Delivery of Health Care"/ (53544)

30. "Referral and Consultation"/ (44989)

31. Health Services Accessibility/ (39281)

32. outpatient clinics, hospital/ (13274)

33. 29 or 30 or 31 or 32 (143984)

34. 28 and 33 (161)

**Figure S1 Logic model of traditional and Patient Initiated Clinic appointment systems**

Eligibility criteria for using the PIC system

Flexible follow-up appointments

Time for appointments increased (guidebook?)

**Traditional appointment system**

**Patient initiated appointment system**

**Table S1 – Characteristics of patient initiated clinics in included studies**

| **Study** | **Written information** | **Telephone help line** | **Initial consultation** | **Annual checkup** |
| --- | --- | --- | --- | --- |
| **Brown (2002)** | √ | √ | × | √ |
| **Sheppard (2009)** | × | √ | × | √ |
| **Kennedy (2003)** | √ (guidebook) | √ | √ | √ (some cases) |
| **Robinson (2001)** | √ (guidebook) | √ | √ | √ (some cases) |
| **Williams (2000)** | × | √ (+ GP contact) | × | √ (24mths) |
| **Hewlett (2000)** | × | √ (+ GP contact) | × | × |
| **Kirwan (2003)** | × | √ | × | √ (24mths) |
| **Hewlett (2005)** | × | √ | × | √ (24mths) |
| **Sands (2009)** | × | √ | √ | × |

**Table S2: Study Quality**

**Key**

**?** = unclear **X** = not reported in study **✓** = reported in study **N/A** = not applicable

**Partial** = only some of the data was reported or applicable

|  | **Brown 2002** | **Hewlett, 2000** | **Hewlett, 2005** | **Kennedy, 2003** | **Kirwan, 2003** | **Robinson,2001** | **Sands, 2009** | **Sheppard, 2009** | **Williams, 2000** |
| --- | --- | --- | --- | --- | --- | --- | --- | --- | --- |
| **Design** | RCT | RCT | RCT | RCT | RCT | RCT | Cross-sectional | RCT | RCT |
| **Eligibility criteria specified** | ✓ | ✓ | ✓ | ✓ | ✓ | ✓ | ✓ | ✓ | ✓ |
| **Power calculation** | X | ✓ | ✓ | ✓ | ✓ | ✓ | X | ✓ | ✓ |
| **Sample size adequate** | ? | ✓ | ? | X | ? | ✓ | ? | X | ? |
| **Adequately randomised** | ✓ | ? | ? | ? | ? | ✓ | N/A | ✓ | ✓ |
| **Allocation concealed** | ? | ? | ? | X | ? | X | N/A | ✓ | X |
| **Baseline details** | ✓ | ✓ | ✓ | ✓ | ✓ | ✓ | ✓ | ✓ | X |
| **Groups similar at baseline** | ✓ | ✓ | ✓ | ✓ | ? | ✓ | ✓ | ✓ | ✓ |
| **Adequately adjusted analysis** | N/A | N/A | X | ? | X | N/A | N/A | ✓ | N/A |
| **Co-interventions** | ✓ | ✓ | ✓ | X | ✓ | ✓ | ? | ✓ | ✓ |
| **Patients blinded to treatment** | N/A | N/A | N/A | N/A | N/A | N/A | N/A | X | N/A |
| **Assessors blinded** | ? | ? | X | X | ? | X | X | X | X |
| **Compliance with treatment** | ✓ | ✓ | ✓ | ? | ✓ | ✓ | ? | ? | ? |
| **Sub group analysis justified** | N/A | N/A | N/A | ✓ | N/A | N/A | N/A | N/A | N/A |
| **Valid measures** | Partial | Partial | Partial | ✓ | Partial | X | Partial | ✓ | X |
| **Reliable measures** | Partial | Partial | Partial | ✓ | Partial | X | Partial | ✓ | X |
| **All participants accounted for** | Partial | ✓ | ✓ | ✓ | ✓ | ✓ | X | ✓ | ✓ |
| **Appropriate analysis** | ✓ | Partial | ✓ | ✓ | Partial | ✓ | ? | ✓ | ✓ |
| **ITT analysis** | X | X | X | ✓ | X | ✓ | X | X | ? |
| **> 80% participants in follow-up** | ✓ | ✓ | X | X | X | ✓ | ✓ | ✓ | ✓ |
| **Conclusions supported by results** | X | ✓ | ✓ | ✓ | ✓ | ✓ | X | ✓ | ? |
| **Inter centre variability** | X | N/A | N/A | ? | N/A | ? | N/A | N/A | ? |

**Appendix 2**

**Data Extraction and Quality Appraisal form**

| Bibliographic details  Reference no:  First author:  Title:  Year:  Citation: | |
| --- | --- |
| STUDY  Country:  Setting:  Recruitment dates:  Study design:  Funding source:  Notes: | INTERVENTION  Description of Intervention:  Description of Comparator:  Notes:  Is the intervention described in sufficient detail for it to be replicated elsewhere? |
| SUBJECTS  Total number:  Inclusion criteria:  Exclusion criteria: |  |
| Patient characteristics   \| \| Mean (SD) \| **control** \| \| **intervention** \| \| \| --- \| --- \| --- \| --- \| --- \| \| N: \|  \| \|  \| \| \| Age *yrs*: \|  \|  \|  \|  \| \| Sex: \|  \|  \|  \|  \| \| M \|  \|  \|  \|  \| \| F \|  \|  \|  \|  \| \|  \|  \|  \|  \|  \| \|  \|  \|  \|  \|  \| \|  \|  \|  \|  \|  \| \| \| --- \| --- \| --- \| --- \| --- \| --- \| --- \| --- \| --- \| --- \| --- \| --- \| --- \| --- \| --- \| --- \| --- \| --- \| --- \| --- \| --- \| --- \| --- \| --- \| --- \| --- \| --- \| --- \| --- \| --- \| --- \| --- \| --- \| --- \| --- \| --- \| --- \| --- \| --- \| --- \| --- \| --- \| --- \| --- \| --- \| --- \| | OUTCOME MEASURES  Primary outcome measure:  Secondary measures:  Method of assessing outcomes:  Length of follow-up:  Notes:  This section should include a list of outcomes measured and the methods of measuring them not the results.  Are the questionnaires validated?  Is the primary outcome measure specified? |

| Results  Total number of participants enrolled:  Number lost to follow-up (if appropriate):   \| \|  \| **control** \| \| **intervention** \| \| \| --- \| --- \| --- \| --- \| --- \| \|  \| n \| mean (sd) \| n \| mean (sd) \| \| *Outcomes* \|  \|  \|  \|  \| \|  \|  \|  \|  \|  \| \|  \|  \|  \|  \|  \| \|  \|  \|  \|  \|  \| \|  \|  \|  \|  \|  \| \|  \|  \|  \|  \|  \| \|  \|  \|  \|  \|  \| \| \| --- \| --- \| --- \| --- \| --- \| --- \| --- \| --- \| --- \| --- \| --- \| --- \| --- \| --- \| --- \| --- \| --- \| --- \| --- \| --- \| --- \| --- \| --- \| --- \| --- \| --- \| --- \| --- \| --- \| --- \| --- \| --- \| --- \| --- \| --- \| --- \| --- \| --- \| --- \| --- \| --- \| --- \| --- \| --- \| --- \| --- \|   Adverse events:  Type of analysis (intention to treat, per protocol):  Results of study analysis (e.g. Dichotomous: odds ratio, risk ratio and confidence intervals, p-value; Continuous: mean difference, confidence intervals):  Notes: |
| --- | --- | --- | --- | --- | --- | --- | --- | --- | --- | --- | --- | --- | --- | --- | --- | --- | --- | --- | --- | --- | --- | --- | --- | --- | --- | --- | --- | --- | --- | --- | --- | --- | --- | --- | --- | --- | --- | --- | --- | --- | --- | --- | --- | --- | --- | --- |
| quality appraisal   \| 1. Study design \| {RCT; X-over trial: CCT; pre-post study} \| \| --- \| --- \| \| 2. Were the study eligibility criteria specified? \| {yes; no; partial} \| \| 3. Was a power calculation performed? \| {yes; no} \| \| 4. Is the sample size adequate? \| {yes; no; unclear} \| \| 5. Is the number randomized stated? \| {yes; no; not applicable} \| \| 6. Is the study properly randomized ┼ \| {yes: no; not applicable; unclear} \| \| 7. Is allocation of treatment concealed? ╪ \| {yes; no; not applicable; unclear} \| \| 8. Are adequate baseline details  escribed? \| {yes; no; partial} \| \| 9. Are groups similar at baseline? \| {yes; no; partial; not applicable} \| \| 10. Are baseline imbalances adequately adjusted for in the analysis? \| {yes; no; not applicable; unclear} \| \| 11. Are similar co-interventions administered? \| {yes; no; unclear; not applicable} \| \| 12. Are patient’s blinded to treatment allocation? \|  \| \| 13. Are outcome assessors blinded? \| {yes; no; unclear} \| \| 14. Is compliance with treatment adequate? \| {yes; no; unclear; not reported} \| \| 15. Were any sub-group analysis justified? \| {yes; no; not applicable} \| \| 16. Were data collection tools shown or known to be valid  for the outcome of interest?± \| {yes; no; unclear} \| \| 17. Were the data collection tools known or were shown to be  consistent and accurate in measuring the outcome of interest?* \| {yes; no; unclear} \| \| 18. Were all study participants accounted for? \| {yes; no} \| \| 19. Are data analyses appropriate? \| {yes; no; partial; unclear} \| \| 20. Is analysis conducted on an ITT basis? \| {yes; no; not applicable} \| \| 21. Are greater than 80% of patients included in the follow-up assessment? \| {yes; no; unclear} \| \| 22. Are the conclusions supported by the results? \| {comment} \| \| 23. Generalisability \| {comment} \| \| 24. Inter-centre variability \| {comment} \| \| 25. General comments \| {comment} \|   ┼ Adequate approaches top sequence generation: computer-generated random numbers, random number tables; inadequate approaches: use of alternation, case record numbers, birth dates or week days  ╪ Adequate approaches to concealment allocation: centrally or pharmacy-controlled randomisation, inadequate approaches: serially numbered envelopes, use of alternation, open random number lists  ± The tools are known to be valid or were shown to measure what they are intended to measure  * The tools are known to be reliable or were shown to be consistent and accurate in measuring the outcome of interest (e.g. test-retest, Cronback’s alpha, interrater reliability) |
| GENERAL COMMENTS  Data extraction performed by: Date:  Data extraction checked by: Date: |

**Appendix 3**

| Psychological and HRQOL Outcomes (results as in original articles) | | | | |
| --- | --- | --- | --- | --- |
| **Study** | **Outcomes** | **Results** | | |
|  |  | **Intervention**  **Mean (Std. Deviation)** | **Standard Care**  **M (SD)** | **comments** |
| Brown, et al. (2002)  BC | *At 12mths*  **EORTC QLQ-C30**   - Physical funct - Role funct - Pain - Dyspnoea - Constipation - Diarrhoea - Cognitive funct - Emotional funct - Social funct - Fatigue - Nausea - Sleep disturb - Loss appetite | 5 (5,8) – 1  2 (2,3) – 0  1 (1,3) – 1  1 (1,3) – 1  1 (1,3) – 0  1 (1,2) – 0  3 (2,4) – 1  5 (4,15) – 3  2 (2,4) – 0  4 (3,9) – 1.5  2 (2,3) – 0  1.5 (1,3) – 1  1 (1,3) – 0 | 6 (5,8) – 2  2 (2,3) – 0  1 (1,3) – 1  1 (1,4) – 1  1 (1,3) – 0  1 (1,2) – 0  3 (2,6) – 1  6 (4,15) – 3.5  2 (2,5) – 0.25  5 (3,8) – 2  2 (2,4) – 0  2 (1,4) – 1  1 (1,2) – 0 | NA |
|  | **HAD**   - Anxiety - Depression | 4 (0,12)  1 (0,7) | 6.5 (0,16)*  2 (0,8)* | *P=0.069  *P=0.232 |
|  | **EORTC QLQ-BR23**   - Arm symptoms - Breast symps - Systemic therapy side eff - Body image - Sexual funct   Future perspect | 3 (3,6) – 1  4 (4,7) – 1  9 (7,13) – 3.25  5 (4,10) – 2.25  2 (2,12) – 4  1 (1,3) – 1 | 4 (3,7) – 2  5 (4,8) – 2  9 (7,14) – 2.5  5 (4,8) – 2  2 (1,12) – 1.5  2 (1,3) – 0 | P=0.024 |
| Sheppard et al. (2009) BC | *At 18mths*  **GHQ (likert)**  Aggregate score  Cases>4 | 22.8  15 (14%) | 23.0  21 (20%) | NA |
|  | FACT-G  FACT Breast  FACT endocrine  FACT B+ES | 81.4  20.1  57.4  158.9 | 81.3  21.8  58.7  161.9 | NA |
|  | Fear | 5.6 | 5.0 | NA |
| Kennedy et al (2003) IBD | *At entrance*  Enablement (after consultation)  Satisfaction with initial consultation | 4.0 (3.9)  65.4 (12.0) | 3.0 (3.9)  62.1 (12.3) | P=0.026  P=0.09 |
|  | *At 12mths*  **IBDQ** score | 172.3 (36.6) | 167.7 (37.5) | P=0.45 |
|  | **SF36** –  Physical functioning  Role limitations physical  Role limitations emotional  Social functioning  Mental health  Energy  Pain  General health perception | 78.1 (25.3)  61.4 (44.1)  72.2 (41.0)  74.8 (31.2)  70.3 (20.9)  51.8 (24.5)  69.5 (27.6)  53.2 (25.1) | 75.8 (26.6)  60.3 (43.2)  72.3 (39.6)  72.2 (29.5)  67.8 (21.3)  48.2 (25.4)  67.1 (23.6)  49.4 (1.8) | P=0.21  P=0.91  P=0.71  P=0.13  P=0.40  P=0.09  P=0.22  P=0.12 |
|  | **HADS** | 11.7 (7.9) | 12.3 (7.6) | P=0.40 |
|  | Satisfaction with hospital visits  Preferred fixed appt  Preferred flexible appt  Changed thought of illness (%yes)  Changed managed illness (%yes)  Changed thought consultant (%yes) | 54.6 (8.5)  25.7%  74.3%  25.0%  20.2%  15.2% | 53.6 (9.1)  40.6%  59.4%  13.2%  6.9%  12.3% | P=0.62  P<0.001  P<0.001 |
|  | Mean **EQ-5D** score | 0.7071 | 0.6909 |  |
| Robinson et al, (2001)  IBD | *At 14 mths*  Acceptability patient  Acceptability clinician | 82% preferred new int  100% pref new int | 95% preferred new int  100% pref new int |  |
|  | IBDQ QOL | 189 | 183 | P=0.16 |
| Williams et al, (2000)  IBD | *At 24mths*  SF36 –   - Physical funct - Role limits (physical probs) - Role limits (emotion probs) - Social funct - Mental health - vitality - bodily pain - General health perception |  | Control better  Control better  Control better  Control better  Control better  Control better  Control better  Control better | *Mean difference*  -3.7 (3.2, -10.5)  -2.7 (11.4, -16.8)  -5.3 (11.4, -22.0)  -0.4 (8.1, -8.9)  -3.7 (2.4, -9.9)  -3.7 (3.3, -10.7)  -2.5 (5.0, -10.0)  -3.5 (2.0, -8.9) |
|  | UKIBDQ – bowel movements/use of fac   - General bowel symptoms - Systemic funct - emotional function - Social function |  | Intervention better  Control better  Intervention better  Control better  Intervention better | 0.3 (7.7, -7.1)  -3.5 (4.0, -10.9)  2.2 (9.9, -5.6)  -1.3 (3.4, -5.9)  0.4 (6.5, -5.7) |
|  | - Patient preference INT - GP preference INT - GP preference CON | 85%  55 patients  15 patients | 41%  53 patients  20 patients |  |
| Hewlett, 2000  (RA) | *At 24 months*  Anxiety | Increased 3% | Increased 11% | NS |
|  | Depression | Increased 4% | Increased 14% | NS |
|  | Self efficacy | 63 | 55 | P=0.053 |
|  | Patient satisfaction  Confidence in system  GP satisfaction  GP confidence (in sys) | 8.56cm  8.5cm  7.66cm  7.46cm | 7.89cm  7.5cm  7.39cm  7.65cm | P<0.05  P<0.05 |
| Kirwan, 2003  (RA) | *Change in scores 0-48mths*  Anxiety | 0.5 | 0.5 |  |
|  | Depression | 0.2 | 0.3 |  |
|  | Helplessness | -0.2 | 1.0 |  |
|  | Self-efficacy – pain  Self-efficacy - function  Self-efficacy – other | 5.1  -1.4  0.4 | 7.4  -7.3  -4.4 |  |
|  | Satisfaction  Confidence | 0.5  0.5 | -0.8  -0.6 | P<0.001  P<0.001 |
| Hewlett, 2005 | *Median change and range at 6 years*  Anxiety | 0 (-2.0 – 3.0) | 0 (-2.0 – 3.0) | P=0.95 |
|  | Depression | 0 (-1.0 – 3.0) | 0 (-1.0 – 2.75) | P=0.80 |
|  | Helplessness | - 1. (-3.0 – 3.0) | 1.0 (-1.75 – 4.0) | P=0.20 |
|  | Self-efficacy – pain  Self-efficacy - function  Self-efficacy – other | 2.0 (12.0 – 16.0)  -2.75 (-15.9 – 5.0)  -3.30 (-11.6 – 8.3) | 1.0 (-10.0 – 19.0)  -6.6 (-20.6 – 2.40)  6.7 (-15.0 – 6.7) | P=0.49  P=0.19  P=0.25 |
|  | Satisfaction  Confidence  GP satisfaction  GP Confidence | 0 (-0.7 – 0.9)  0.15 (-0.73 – 0.43)  8.4 (7.5 – 9.6)  8.4 (7.25 – 9.45) | 1.1 (-2.70 – 0.25)  -1.0 (-2.35 – 0.20)  7.5 (5.5 – 8.57)  8.0 (5.72 – 8.7) | P=0.0004  P=0.0005  P=0.005  P=0.04 |
| Sands, 2009  (RA) | QOL AIMS2-SF score | 19.25 (7.79) | 18.71 (7.33) | P=0.746 |
|  | Anxiety and Depression (HADS score) | 14.78 (8.10) | 14.54 (7.73) | P=0.890 |
|  | P satisfaction  P confidence | 7.15 (3.41)  7.35 (3.23) | 7.17 (3.02)  7.39 (2.85) | P=0.990  P=0.995 *PLUS* |
